# Combination therapy of Sanjie Zhentong capsules for endometriosis: Impact on hormone regulation and serum expression of FOLR1 and MSLN

**DOI:** 10.1097/MD.0000000000039953

**Published:** 2024-10-11

**Authors:** Xiaojuan Cheng, Yibing Lan, Linyun Zhang, Huaquan Cheng

**Affiliations:** a Gynecological Endocrinology Department, Zhejiang Huzhou Anji Maternal and Child Health Hospital, Huzhou, Zhejiang, China; b Gynecology, Zhejiang University Affiliated Hospital of Obstetrics and Gynecology, Hangzhou, Zhejiang, China; c Department of Chinese Medicine, Zhejiang Anji People’s Hospital Branch, Anji, Zhejiang, China.

**Keywords:** endometriosis, pain, Sanjie Zhentong capsules, sex hormones, uterine artery blood flow

## Abstract

Examining the impact of Sanjie Zhentong capsules on hormone levels and serum expression of folate receptor 1 (FOLR1) and Mesothelin (MSLN) in endometriosis patients. A cohort of 150 endometriosis patients admitted between June 2019 and June 2023 was divided into 2 groups based on Sanjie Zhentong intake, comprising 75 cases each. Comparative analyses encompassed uterine artery hemodynamic parameters, ultrasound (B-mode) findings, Visual Analog Scale scores, traditional Chinese medicine symptom scores, Endometriosis Health Profile-30 scores, and serum levels of FOLR1, MSLN, and sex hormones before and after treatment. Post-treatment, both groups exhibited increased peak systolic velocity compared to pretreatment, with the Sanjie Zhentong group showing a significantly higher increase (*P* < .05). Moreover, resistance index decreased in both groups post-treatment, with the Sanjie Zhentong group displaying a greater reduction (*P* < .05). Adnexal mass diameters, endometrial thickness, and uterine volume decreased post-treatment in both groups, with the Sanjie Zhentong group experiencing a more pronounced decrease (*P* < .05). Serum FOLR1, MSLN, and sex hormone levels also decreased post-treatment in both groups, with the Sanjie Zhentong group exhibiting a more significant decrease (*P* < .05). Additionally, pain scores, traditional Chinese medicine symptoms, and Endometriosis Health Profile-30 scores decreased post-treatment in both groups, with the Sanjie Zhentong group demonstrating a greater reduction (*P* < .05). Sanjie Zhentong capsules demonstrate efficacy in regulating sex hormones, reducing serum FOLR1 and MSLN expression, alleviating pain, shrinking adnexal masses, decreasing uterine size, and improving uterine artery blood flow in endometriosis patients.

## 1. Introduction

Endometriosis is a gynecological benign disease that is estrogen-dependent and prevalent among women of reproductive age.^[1]^ Folate receptor 1 (FOLR1) can influence the development of gynecological malignancies and epithelial-mesenchymal transition (EMT) through pathways such as regulating folate metabolism and estrogen receptor expression. Mesothelin (MSLN) is a glycoprotein that is overexpressed in malignant pleural mesothelioma and in patients with EMT.^[2]^ Drospirenone and ethinylestradiol tablets are commonly used Western medicine treatments for endometriosis; they can alleviate symptoms of dysmenorrhea and reduce lesion size, but their overall efficacy is limited.^[3]^ The operation is an invasive operation, and the cost is high, and the patient ‘s acceptance is poor. Most patients still receive conservative treatment.

In traditional Chinese medical texts, there is no specific disease term like “EMT.” Based on clinical presentations, it can be categorized under terms such as “dysmenorrhea” and “Zhengjia.” These conditions are often associated with poor circulation of qi and blood, the presence of phlegm and turbidity, and stagnation of stagnant blood in the uterus. Over time, these conditions can lead to the development of “Zhengjia.” The treatment approach typically involves promoting blood circulation, dispelling blood stasis, and resolving masses.^[4]^ Sanjie Zhentong capsules are a commonly used traditional Chinese herbal remedy for conditions such as dysmenorrhea, pelvic masses, and irregular menstruation. They are believed to have the effects of promoting blood circulation, dispelling blood stasis, and relieving pain.^[5]^ However, there have been no reported studies on their effects on hormone levels, as well as the expression of serum FOLR1 and MSLN in patients. The aim of this study is to observe the therapeutic efficacy of Sanjie Zhentong capsules in the treatment of EMT and evaluate their impact on patients’ hormone levels, as well as the expression of serum FOLR1 and MSLN.

## 2. Materials and methods

### 2.1. Inclusion and exclusion criteria

Inclusion criteria: (1) in line with the “guidelines for the diagnosis and treatment of endometriosis”^[6]^ in the standard, combined with symptoms, gynecology and imaging diagnosis. (2) Patients with regular menstruation aged 18 to 45 years. (3) In line with the “EMT Integrated Traditional Chinese and Western Medicine Diagnosis and Treatment Guidelines”^[7]^ in the phlegm and blood stasis type standard. (4) Not taking any hormone medications that may affect test results within the past 6 months, including estrogen medications, progesterone medications, and ovulation-inducing medications, was required for eligibility. (5) Can be followed up. (6) No history of surgical treatment of uterine appendages. (7) The patient himself signed the consent form.

Exclusion criteria: (1) dysmenorrhea, infertility or pelvic mass, nodules caused by other reasons. (2) With important organ dysfunction. (3) Recent fertility requirements. (4) Reproductive organ cancer or other malignant tumors. (5) Combined with adenomyosis or uterine fibroids. (6) With mental illness or hematopoietic, immune and endocrine metabolic diseases. (7) There may be a history of severe infection, fungal infection, tuberculosis, etc. (8) Withdrawal due to personal will during treatment. (9) Other special conditions occurred in the middle, and the treatment was stopped or the medication regimen was changed. (10) Poor compliance, referring to individuals who, due to various reasons, are unable to cooperate with follow-up assessments.

### 2.2. General data

This study is a retrospective study approved by the Ethics Committee of Anji Maternal and Child Health Hospital in Huzhou City, Zhejiang Province. In this study, 75 patients with endometriosis who had previously taken three-section analgesics from June 2019 to June 2023 were selected as the experimental group, and 75 patients with 1-to-1 matching endometriosis who had not previously taken three-section analgesics were selected as the control group, with a total of 150 cases. Sanjie Zhentong group (75 cases) took norspirosterone and ethinylestradiol tablets on the basis of Sanjie Zhentong capsule, control group (75 cases) took norspirosterone and ethinylestradiol tablets on the basis of norspirosterone and ethinylestradiol tablets. The purpose of matching was to compare the general data of the 2 groups, excluding the influence of confounding factors, and the basic information of the 2 groups was consistent except for the difference in treatment style (*P* > .05).

### 2.3. Methods

The control group was treated with drospirenone and ethinylestradiol tablets (production unit: Abbott Company Co., Ltd., specifications: 3 mg of drospirenone and 0.03 mg of ethinylestradiol per tablet), starting on the 5th day of menstruation, 1 tablet/time, 2 times/day, total 20 days, and discontinued until the next menstrual cycle.

The Sanjie Zhentong capsule group was treated with Sanjie Zhentong capsule (production unit: Jiangsu Kangyuan Pharmaceutical Co., Ltd., specification: 0.4 g) on the basis of drospirenone and ethinylestradiol tablets. The usage and dosage of drospirenone and ethinylestradiol tablets were the same as those of the control group. Oral Sanjie Zhentong capsule 4 capsules/time, 3 times/d, discontinued during menstruation. Both groups were treated continuously for 6 menstrual cycles. The composition of Sanjie Zhentong Capsules is as shown in Table 1.

**Table 1:** The composition of Sanjie Zhentong capsule.

Ingredients	Quality
Dragon blood	62 g
Panax Notoginseng	62 g
Zhebei Bulbus Fritillariae Thunbergii	100 g
Coix Seed	176 g

### 2.4. Observed indexes

(1) Uterine artery hemodynamic parameters: before treatment and after 6 menstrual cycles of treatment, the uterine artery hemodynamic parameters of the 2 groups were detected by ultrasound, including peak systolic velocity (PSV) and resistance index (RI). The instrument is Philips IE33 color Doppler ultrasound diagnostic instrument.
(2) B-ultrasound examination: before and after 6 menstrual cycles of treatment, ultrasound was used to measure the diameters of adnexal masses, endometrial thickness, and uterine volume in both groups. The instrument used for this purpose was the Philips IE33 color Doppler ultrasound diagnostic device.
(3) Serum factors: before treatment and after 6 menstrual cycles of treatment, 5 mL of fasting venous blood samples were collected from both groups of patients in the morning. The samples were left at 4 °C for half an hour and then centrifuged at a speed of 3000 rpm for 10 minutes. The serum was collected and stored for further analysis. Serum FOLR1 and MSLN were detected using the ELISA method with kits provided by Wuhan Bosideng Biotechnology Co., Ltd., and the instrument used was the Shandong Bokang BK-EL10A enzyme-linked immunosorbent assay analyzer. Additionally, serum levels of estradiol (E2), progesterone (P), luteinizing hormone (LH), and follicle-stimulating hormone (FSH) were measured using the chemiluminescent immunoassay method with kits provided by Nanjing Senbeijia Biotechnology Co., Ltd., and the instrument used was the Shandong Bokang fully automatic biochemical analyzer.
(4) Visual Analogue Scale (VAS)^[8]^: scoring on a scale of 10 points: in clinical practice, 0 points indicate no pain, 1 to 3 points indicate mild pain, and 4 to 6 points and 7 to 10 points indicate moderate and severe pain, respectively. Traditional Chinese Medicine Symptom Score: main symptoms include lower abdominal pain, dyspareunia, nodules, and a feeling of rectal fullness. The severity of these symptoms is scored from 0 to 6 points, with higher scores indicating more severe symptoms. Secondary symptoms include menstrual irregularities with blood clots, increased vaginal discharge, dizziness, nausea with phlegm, heavy limbs, sticky and uncomfortable stools, or diarrhea. The severity of these symptoms is scored from 0 to 3 points, with higher scores indicating more severe symptoms. Endometriosis Health Profile-30 (EHP-30) score: this questionnaire covers 5 dimensions with a total of 30 items. Higher scores indicate lower quality of life.

### 2.5. Statistical processing

The data were collected and sorted out by Execl table, and processed by SPSS25.0 software. The measurement data of this study were tested by ShaPiro–Wilk normal distribution test, and the measurement data were described by ($\bar{X}\pm s$). The independent sample *t* test was used to compare the groups, and the paired *t* test was used to compare the groups. If it did not conform to the normal distribution, it was expressed by M (P25–P75). The rank sum test was used for comparison between groups, and the count data was described by n, and the chi-square test was used for comparison.

## 3. Results

### 3.1. Comparison of 2 groups of general data

Two groups of general data were compared (*P* > .05). See Table 2.

**Table 2:** Comparison of 2 groups of general data.

General information	Control group	Sanjie analgesia group	χ^2^/t	*P*
Age (years)	35.85 ± 4.45	36.02 ± 4.19	0.241	.810
BMI (kg/m^2^)	23.47 ± 2.05	23.35 ± 2.27	0.340	.735
Duration of disease (years)	3.06 ± 0.78	2.99 ± 0.85	0.525	.600
Marital status (example)			0.502	.479
Be married	50 (66.67)	54 (72.00)		
Spinster	25 (33.33)	21 (28.00)		
Pregnancy number (frequency)	2.05 ± 0.56	2.12 ± 0.54	0.779	.437
Menstrual cycle (d)	30.58 ± 2.14	30.64 ± 2.57	0.155	.877
R-AFS staging (example)			0.469	.791
Phase I	27 (36.00)	24 (32.00)		
Phase II	28 (37.33)	32 (42.67)		
Phase III	20 (26.67)	19 (25.33)		
Induced abortion history (example)			1.799	.180
Yes	25 (33.33)	33 (44.00)		
No	50 (66.67)	42 (56.00)		

### 3.2. Comparison of uterine artery hemodynamic parameters between the 2 groups

The uterine artery hemodynamic parameters of the 2 groups were compared before treatment (*P* > .05). The PSV of the 2 groups increased after treatment, and the analgesic group was higher than that of the control group (*P* < .05). The RI of the 2 groups decreased after treatment, and the RI of the Sanjie analgesia group was lower than that of the control group (*P* < .05). See Table 3.

**Table 3:** Comparison of uterine artery hemodynamic parameters between the 2 groups.

Group	PSV (cm/s)		RI	
	Before medication	After medication	Before medication	After medication
Control group	42.12 ± 3.89	45.13 ± 4.05*	0.89 ± 0.18	0.71 ± 0.13*
Sanjie analgesia group	41.94 ± 4.08	48.74 ± 4.23*	0.90 ± 0.15	0.62 ± 0.10*
t	0.277	5.339	0.370	4.752
*P*	.783	.000	.712	.000

* There were differences before and after medication.

### 3.3. Comparison of adnexal mass diameter, endometrial thickness, and uterine volume between the 2 groups

The adnexal mass diameter, endometrial thickness, and uterine volume in both groups were compared before medication (*P* > .05). However, after medication, these parameters decreased in both groups, with the Sanjie Zhentong group showing a greater reduction compared to the control group (*P* < .05). The endometrial thickness of the 2 groups increased after treatment, and the Sanjie Zhentong group was higher than the control group after treatment (*P* < .05). See Table 4.

**Table 4:** Comparison of adnexal mass diameter, endometrial thickness, and uterine volume between the 2 groups.

Group	Diameter of attachment package block (cm)		Endometrial thickness (mm)		Uterine volume (cm^3^)	
	Before medication	After medication	Before medication	After medication	Before medication	After medication
Control group	6.08 ± 1.74	4.86 ± 1.23*	6.85 ± 1.45	7.54 ± 1.03*	138.52 ± 20.56	119.82 ± 16.42*
Sanjie analgesia group	6.13 ± 1.68	3.12 ± 0.97*	6.76 ± 1.56	8.36 ± 0.91*	137.74 ± 23.69	102.77 ± 14.73*
*t*	0.179	9.620	0.366	5.167	0.215	6.694
*P*	.858	.000	.715	.000	.830	.000

* There were differences before and after medication.

### 3.4. Comparison of serum FOLR1 and MSLN between the 2 groups

Serum FOLR1 and MSLN were compared between the 2 groups before treatment (*P* > .05). The levels of serum FOLR1 and MSLN in the 2 groups were lower than those before treatment, and those in the Sanjie Zhentong group were lower than those in the control group (*P* < .05). See Table 5.

**Table 5:** Comparison of serum FOLR1 and MSLN between the 2 groups.

Group	FOLR1 (pg/mL)		MSLN (µg/L)	
	Before medication	After medication	Before medication	After medication
Control group	304.85 ± 85.69	182.23 ± 34.45*	9.96 ± 2.24	5.23 ± 1.77*
Sanjie analgesia group	299.74 ± 90.56	156.21 ± 26.02*	10.02 ± 2.03	4.45 ± 1.16*
*t*	0.355	5.220	0.172	3.192
*P*	.723	.000	.864	.002

* There were differences before and after medication.

### 3.5. Comparison of serum sex hormones between the 2 groups

Serum hormone levels were compared between the 2 groups before medication (*P* > .05). However, after medication, both groups exhibited a decrease in serum levels of E2, P, LH, and FSH, with the Sanjie Zhentong group showing a greater reduction compared to the control group (*P* < .05). See Table 6.

**Table 6:** Comparison of serum sex hormones between the 2 groups.

Group	E2 (pmol/L)		P (nmol/L)		LH (U/L)		FSH (U/L)	
	Before medication	After medication	Before medication	After medication	Before medication	After medication	Before medication	After medication
Control group	262.02 ± 64.27	155.23 ± 43.21	30.47 ± 8.56	20.25 ± 4.74	8.85 ± 2.03	5.05 ± 1.54	5.69 ± 1.21	4.15 ± 0.97
Sanjie analgesia group	256.98 ± 70.11	136.74 ± 38.52	30.12 ± 9.17	16.41 ± 4.08	8.91 ± 1.89	3.86 ± 1.27	5.63 ± 1.18	3.24 ± 0.86
*t*	0.459	2.766	0.242	5.317	0.187	5.163	0.307	6.079
*P*	.647	.006*	.809	.000*	.852	.000*	.759	.000*

* There were differences before and after medication.

### 3.6. Comparison of VAS scores, traditional Chinese medicine symptom scores, and EHP-30 scores between the 2 groups

VAS scores, traditional Chinese medicine symptom scores, and EHP-30 scores were compared between the 2 groups before medication (*P* > .05). However, after medication, both groups exhibited a decrease in VAS scores, traditional Chinese medicine symptom scores, and EHP-30 scores, with the Sanjie Zhentong group showing a greater reduction compared to the control group (*P* < .05). See Table 7. Traditional Chinese medicine symptom scores are presented in Table S1, Supplemental Digital Content, http://links.lww.com/MD/N664.

**Table 7:** Comparison of VAS scores, traditional Chinese medicine symptom scores, and EHP-30 scores between the 2 groups.

Group	VAS score (points)		TCM symptom score (points)		EHP-30 score (points)	
	Before medication	After medication	Before medication	After medication	Before medication	After medication
Control group	6.52 ± 1.04	2.52 ± 0.67*	28.47 ± 3.23	13.74 ± 1.96*	38.85 ± 5.46	21.89 ± 3.61*
Sanjie analgesia group	6.47 ± 1.11	1.71 ± 0.43*	29.01 ± 3.11	9.65 ± 1.44*	39.07 ± 5.14	16.24 ± 3.07*
*t*	0.285	8.811	1.043	14.564	0.254	10.325
*P*	.776	.000	.299	.000	.800	.000

* There were differences before and after medication.

### 3.7. Adverse reactions in both groups

No severe adverse reactions were observed, and the patients’ liver and kidney functions, as well as electrocardiograms, showed no abnormalities.

## 4. Discussion

EMT involves the implantation and growth of endometrial tissue outside the uterine cavity, such as in the ovaries, uterosacral ligaments, and other pelvic areas. The exact mechanisms behind its development are not fully understood, but existing research suggests it may be related to factors such as immune-inflammatory responses, abnormal hormonal levels, genetic mutations, among others.^[9]^ Despite being classified as a benign condition, endometriosis exhibits characteristics of proliferation, infiltration, metastasis, and recurrence, resembling malignant behaviors. Therefore, once diagnosed, proactive treatment is recommended.^[10]^ Drospirenone and ethinylestradiol is a short-acting contraceptive. Because it can inhibit the secretion of gonadotropin by pituitary and inhibit the proliferation activity of endometrial cells, it is also used in the treatment of EMT, which is helpful to alleviate the symptoms of dysmenorrhea.^[11]^

According to traditional Chinese medicine theory, endometriosis lesions are considered as blood stagnation that has strayed from the meridians, causing blockage in the uterine cavity, leading to impaired qi and blood circulation, and where there is stagnation, there is pain. Long-term emotional distress, cold retention, and energy stagnation can all contribute to blood stasis.^[12]^ The formula of Sanjie Zhentong capsules is refined and, in this formula, Dragon Blood Resin plays the lead role. It excels at promoting blood circulation, alleviating blood stasis, and relieving pain. Notoginseng acts as the secondary herb, proficient in enhancing blood flow, stopping bleeding, reducing inflammation, and relieving pain. It is complemented by Zhe Bei Mu for reducing hyperthermia and clearing mucus, as well as reducing inflammation; Yi Yi Ren for promoting fluid metabolism, draining pus, detoxifying, and reducing fibrous masses. All these herbs work together to achieve the effects of breaking up blood stasis, eliminating blockages, and relieving pain.^[13]^ Yin et al^[14]^ found that Sanjie Zhentong Capsule combined with drospirenone and ethinylestradiol was effective in the treatment of EMT, which was helpful to alleviate clinical symptoms and reduce the size of lesions and uterus. However, the study did not explore its mechanism of action in depth.

This study found that both groups showed a reduction in VAS scores, TCM symptom scores, and EHP-30 scores after treatment, with the Sanjie Zhentong group showing a greater reduction compared to the control group. This result suggests that Sanjie Zhentong capsules can alleviate pain symptoms and traditional Chinese medicine symptoms in EMT patients, improving their overall quality of life. This outcome aligns with previous research conclusions.^[15]^ The effectiveness of Sanjie Zhentong capsules may be attributed to the presence of components such as Dragon Blood Resin B and Taspine in Dragon Blood Resin, which can accelerate venous and lymphatic drainage, promote the absorption of inflammatory substances, inhibit platelet aggregation, prevent thrombus formation, and enhance local metabolism.^[16]^ The saponin components found in Sanqi can dilate blood vessels, possess antiplatelet effects, and exhibit pharmacological actions such as anti-inflammation, analgesia, and anti-fatigue.^[17]^ The alkaloids present in Zhebei Mu have spasmolytic and central analgesic effects.^[18]^ The alkaloid compounds in Yi Yi Ren have antipyretic, analgesic, and sedative properties. Coix Seed polysaccharides can regulate immune function and have anti-tumor effects, while Coix Seed esters can inhibit the proliferation of tumor cells.^[19]^

The increase in uterine volume in EMT is due to the growth of lesions, and the delayed uterine artery hemodynamics in these patients may be a primary cause of their infertility.^[20]^ This study found that both groups had an increase in PSV after treatment, with the San Jie Zhen Tong capsule group having a higher increase compared to the control group. Both groups showed a decrease in RI after treatment, with the San Jie Zhen Tong capsule group showing a greater reduction compared to the control group. The diameter of the adnexal mass, endometrial thickness, and uterine volume decreased in both groups after treatment, with the San Jie Zhen Tong capsule group showing a greater reduction. These results suggest that San Jie Zhen Tong capsules can reduce the diameter of masses and the size of the uterus in EMT patients, thereby improving uterine artery blood flow. This is attributed to the pharmacological effects of San Jie Zhen Tong capsules, such as vasodilation, as seen with Dragon Blood Resin and Panax Notoginseng, which promote blood circulation. Additionally, the anti-inflammatory properties of Zhe Bei Mu and the anti-inflammatory and analgesic properties of Yi Yi Ren contribute to reducing the size of lesions and uterine volume.

The growth of EMT lesions is related to sex hormones, and patients with EMT have higher levels of sex hormones in their bodies than normal. FOLR1 is a member of the receptor family that can take up a large amount of folic acid from plasma, promoting cell proliferation by regulating cell growth control and signal transduction.^[21]^ MSLN is a surface antigen on tumor cells that has a promoting effect on processes such as tumor cell growth, invasion, and metastasis.^[22]^ Both FOLR1 and MSLN are associated with the malignant behavior of EMT lesions, including proliferation, infiltration, metastasis, and recurrence.^[23]^ This study found that the levels of serum FOLR1, MSLN, and sex hormones decreased after medication in both groups, with a greater reduction observed in the analgesic capsule group compared to the control group. This result suggested that the analgesic capsules can regulate sex hormones in patients with EMT and reduce the expression of serum FOLR1 and MSLN. This is one of the important mechanisms of its treatment for EMT. However, it is not yet clear which specific drug components in the analgesic capsules are responsible for the pharmacological effects mentioned above, and further research is needed in the future to explore this.

In summary, the analgesic capsules can regulate sex hormones in patients with endometriosis, reduce the levels of serum FOLR1 and MSLN, alleviate pain, decrease the size of nodules, improve uterine size, and enhance blood flow in the uterine arteries.

## Author contributions

**Conceptualization:** Xiaojuan Cheng, Yibing Lan, Linyun Zhang, Huaquan Cheng.

**Data curation:** Xiaojuan Cheng, Yibing Lan, Linyun Zhang, Huaquan Cheng.

**Formal analysis:** Yibing Lan, Linyun Zhang, Huaquan Cheng.

**Investigation:** Xiaojuan Cheng, Linyun Zhang.

**Methodology:** Xiaojuan Cheng, Linyun Zhang.

**Supervision:** Linyun Zhang, Huaquan Cheng.

**Validation:** Yibing Lan.

**Writing – original draft:** Xiaojuan Cheng, Yibing Lan, Huaquan Cheng.

**Writing – review & editing:** Xiaojuan Cheng, Yibing Lan.

## Supplementary Material


