# Supplementary material for: Combination therapy of Sanjie Zhentong capsules for endometriosis: Impact on hormone regulation and serum expression of FOLR1 and MSLN
Source: Medicine (Baltimore). 2024 Oct 11;103(41):e39953. doi: 10.1097/MD.0000000000039953 (PMC11479455; doi:10.1097/MD.0000000000039953)
Supplement: Supplementary file 1 [file medi-103-e39953-s001.doc]

| Chief Complaint | 0 points | 2 points | 4 points | 6 points |
| --- | --- | --- | --- | --- |
| Dysmenorrhea | None | Mild abdominal pain during or after menstruation, intermittent, not affecting normal activities | More severe abdominal pain during or after menstruation, unable to perform normal daily activities. | Severe abdominal pain during or after menstruation, unable to get up, requiring bed rest. |
| Secondary Complaint | 0 points | 1 points | 2 points | 3 points |
| Blood Clots | None | Occasional small blood clots | Moderate-sized blood clots occasionally observed | Large blood clots frequently observed |
| Menstrual Dark Color | None | Menstrual blood color slightly dark | Menstrual blood color dark red | Menstrual blood color dark and almost black |
| Lumbago and Weakness of the Loin and Knee | None | Occasional minor discomfort, short duration | Symptoms are noticeable and interfere with normal activities | Symptoms are persistent, severe, intolerable, and significantly interfere with daily life |
| Dizziness and Tinnitus | None | Occasionally occurs, short duration, does not affect daily life | Symptoms are noticeable, intermittent, tolerable | Symptoms are noticeable, intermittent, tolerable |
| Fatigue and Weakness | None | Occasionally feels slight fatigue and weakness | Symptoms frequently occur, tolerable | Symptoms are persistent, severe, intolerable |
| Facial Dullness | None | Facial complexion slightly yellowish lacking luster | Facial complexion yellowish lacking luster | Facial complexion dark lacking luster |
| Tongue Appearance | Normal | Tongue is normal without ecchymosis or petechiae | Tongue is dull with visible petechiae | Tongue is dark purple with ecchymosis |
| Pulse Examination | Normal | Pulse is slightly sunken or wiry, thin | Pulse is sunken, with a thin or wiry quality | Pulse is sunken, wiry, and thin |

Supplementary Table 1: Traditional Chinese Medicine Syndrome Scoring Scale
